# Airflow Dynamics of Coughing in Healthy Human Volunteers by Shadowgraph Imaging: An Aid to Aerosol Infection Control

**DOI:** 10.1371/journal.pone.0034818

**Published:** 2012-04-20

**Authors:** Julian W. Tang, Andre Nicolle, Jovan Pantelic, Gerald C. Koh, Liang De Wang, Muhammad Amin, Christian A. Klettner, David K. W. Cheong, Chandra Sekhar, Kwok Wai Tham

**Affiliations:** 1 Department of Laboratory Medicine, National University Hospital, Queenstown, Singapore; 2 Department of Building, School of Design and Environment, National University of Singapore, Queenstown, Singapore; 3 Saw Swee Hock School of Public Health, National University of Singapore, Queenstown, Singapore; Duke-NUS Graduate Medical School, Singapore

## Abstract

Cough airflow dynamics have been previously studied using a variety of experimental methods. In this study, real-time, non-invasive shadowgraph imaging was applied to obtain additional analyses of cough airflows produced by healthy volunteers. Twenty healthy volunteers (10 women, mean age 32.2±12.9 years; 10 men, mean age 25.3±2.5 years) were asked to cough freely, then into their sleeves (as per current US CDC recommendations) in this study to analyze cough airflow dynamics. For the 10 females (cases 1–10), their maximum detectable cough propagation distances ranged from 0.16–0.55 m, with maximum derived velocities of 2.2–5.0 m/s, and their maximum detectable 2-D projected areas ranged from 0.010–0.11 m^2^, with maximum derived expansion rates of 0.15–0.55 m^2^/s. For the 10 males (cases 11–20), their maximum detectable cough propagation distances ranged from 0.31–0.64 m, with maximum derived velocities of 3.2–14 m/s, and their maximum detectable 2-D projected areas ranged from 0.04–0.14 m^2^, with maximum derived expansion rates of 0.25–1.4 m^2^/s.

These peak velocities were measured when the visibility of the exhaled airflows was optimal and compare favorably with those reported previously using other methods, and may be seen as a validation of these previous approaches in a more natural setting. However, the propagation distances can only represent a lower limit due to the inability of the shadowgraph method to visualize these cough airflows once their temperature cools to that of the ambient air, which is an important limitation of this methodology.

The qualitative high-speed video footage of these volunteers coughing into their sleeves demonstrates that although this method rarely completely blocks the cough airflow, it decelerates, splits and redirects the airflow, eventually reducing its propagation. The effectiveness of this intervention depends on optimum positioning of the arm over the nose and mouth during coughing, though unsightly stains on sleeves may make it unacceptable to some.

## Introduction

Since the severe acute respiratory syndrome (SARS) outbreaks of 2003, there has been a great interest in developing methods to assess the risks for the aerosol (or airborne) transmission of human infectious agents, such as the various subtypes (e.g. seasonal H3N2, avian H5N1, pandemic H1N1) of influenza A viruses [1]. Many of these have involved the sampling of air directly from various indoor healthcare environments [2]–[4] or human volunteers [5]–[7]. However, there have been relatively few studies examining the airflow dynamics of more specific, natural human respiratory activities, such as breathing, talking, laughing, coughing and sneezing [8], which provide the main driving forces for the expulsion of saliva or mucus droplets in human-generated aerosols that may be carrying a variety of infectious agents transmissible via the airborne route [9].

The specific use of the schlieren and shadowgraph techniques for clinical imaging have been recently revived [10]–[13], but such schlieren photography has been used for the visualization of human-generated airflows since the 1970s as comprehensively reviewed by Clark and de Calcina-Goff [14]. The schlieren and shadowgraph airflow visualization method has the advantage of not using any irritant or toxic tracers, or intense (e.g. laser) lighting [13]. Only a spherical concave high-precision mirror with a relatively low voltage white (e.g. LED) light source is required. This has the great advantage of allowing the use of human volunteers who can perform various respiratory activities in front of the mirror to allow realistic airflow patterns to be visualized and recorded for further analysis [8], [12].

In this study, the shadowgraph approach has been used to investigate the specific airflow patterns produced from coughing by healthy volunteers. Qualitative video images of the same volunteers coughing into their sleeves, as recommended by the US Centers for Disease Control and Prevention (CDC), Atlanta, USA (http://www.cdc.gov/flu/protect/covercough.htm), are also presented to assess the effectiveness of this technique in limiting the dissemination of aerosols that may be carrying infectious agents.

## Methods

### Imaging set-up

**Figure 1:**
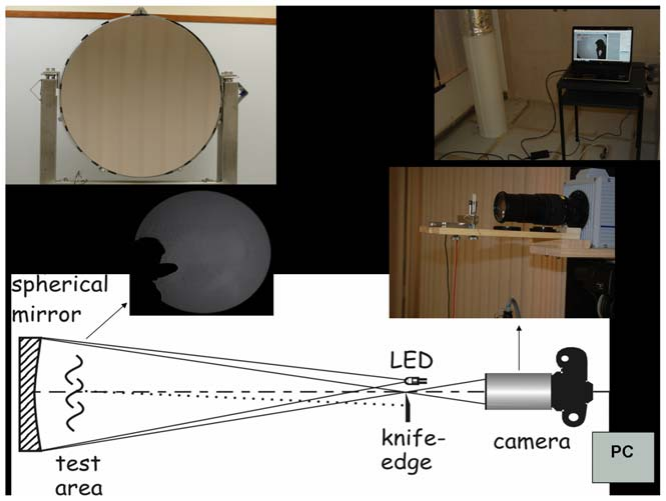
Experimental set-up of the shadowgraph imaging system. Schematic of the layout with the large, 1-m diameter, spherical concave f/5 mirror and subject test area at one end, and the high-speed camera with the LED light-source and the image capture system (laptop) approximately 10 m away at the other end of the environmental chamber. Note that the schematic diagram has been shown previously to describe this experimental set-up [8].

The shadowgraph imaging system used in this study has been described in detail elsewhere [8]. Briefly, a large 1 m diameter, high precision (astronomical quality), spherical concave mirror of 10 m radius (i.e. of focal length of 5 m, an aperture of f/5, Cosmo Optics, Inc., Middletown, NY, USA) was used to reflect light produce by a white LED light source positioned at its centre of curvature, which was 1.6 m above ground level. This height was selected as it would allow the image of the head of most people of average height to be captured in the mirror. Immediately behind the LED was a high-speed digital camera (Photron SA1.1 camera, Dynamic Analysis System, Pte Ltd, Singapore) with a 70–300 mm ED Nikkor lens (Nikon Inc., Melville, NY), which captures and records the shadowgraph images produced by the reflected LED light from the mirror when a human subject is standing approximately 1 m in front of the mirror (Figure 1). Frame-rates of 500–200 frames per second (fps) were used in this study at the 1024×1024 maximum pixel resolution permitted with this high-speed camera (no audio recording is possible with this camera).

Airflow ‘shadowgraph’ images are produced when reflected light from the mirror is refracted to different degrees as it passes through the warmer (less dense) exhaled air (around 30°C) of the human subject as it mixes with the cooler (denser) air in the experimental laboratory (ambient air temperature 18–20°C, relative humidity 60% during this study) [8]. Smaller temperature differences than this (e.g. when the laboratory temperature rose to 24°C or higher) produced images with less contrast that were difficult to analyze, suggesting that a temperature difference of at least 10°C was optimal for this visualization system. The focus of the black-and-white shadowgraph image was a balance between defocusing the camera lens to obtain enough shadowgraph of the cough airflows, yet keeping enough image sharpness to allow the boundaries of these airflows to be defined sufficiently well for digital analysis later. These black-and-white shadowgraph images were found to offer a better black-and-white contrast and ‘definable edges’ for the visible airflow boundaries.

These shadowgraph images were downloaded from the camera into a laptop (and other large capacity, digital data storage hard drives) after each imaging experiment for further analysis. The same laptop was also used to control the high-speed camera remotely using proprietary software (Photron Fastcam Viewer Ver.325, freely available from: http://www.photonicsonline.com/article.mvc/Photrons-Fastcam-Viewer-Software-Features-0002).

### Human volunteers

#### Ethics statement

Ethics approval for this study using human volunteers was granted by the Domain Specific Review Board of the National Healthcare Group/National University Health System (DSRB reference no. E/09/024). All volunteers participating in this study gave both written and verbal consent.

Twenty healthy human volunteers with no acute or chronic respiratory illness were recruited for the cough study. All volunteers were over 21 years of age and mostly were either staff or graduate students of the National University Hospital or the National University of Singapore respectively. Recruited volunteers received a small cash reimbursement for their time and inconvenience. Each volunteer was asked for their height and weight in order to calculate their body-mass index (BMI), as well as their smoking status.

For capturing their cough images, each volunteer was asked to cough freely (at least two bouts) across the mirror as a control. They were then asked to cover their mouth and nose with their arm and repeat the coughing (as suggested on the US CDC website: http://www.cdc.gov/flu/protect/covercough.htm) in order to visualize the airflow patterns produced. The ‘free’ coughs (i.e. those coughs that were not covered by the arm) were also used for the estimates of propagation distance and velocity, and maximum 2-dimensional (2-D) projected area covered over time.

No specific posture was requested of the volunteers. They were asked to just perform their coughs in their usual manner.

### Analysis of recorded images

The raw images from the Photron high-speed camera were recorded as individual TIFF files. For the video montages, these were saved using the proprietary camera/image analysis software PFV (Photron Fastcam Viewer) then converted to smaller and more manageable JPEG files for editing. Final presentations were further edited and annotated using Corel VideoStudio Pro X3 (Corel Corp., Ottawa, Canada) and Windows Movie Maker v.5.1 (Microsoft Corp., Redmond, WA, USA).

**Figure 2:**
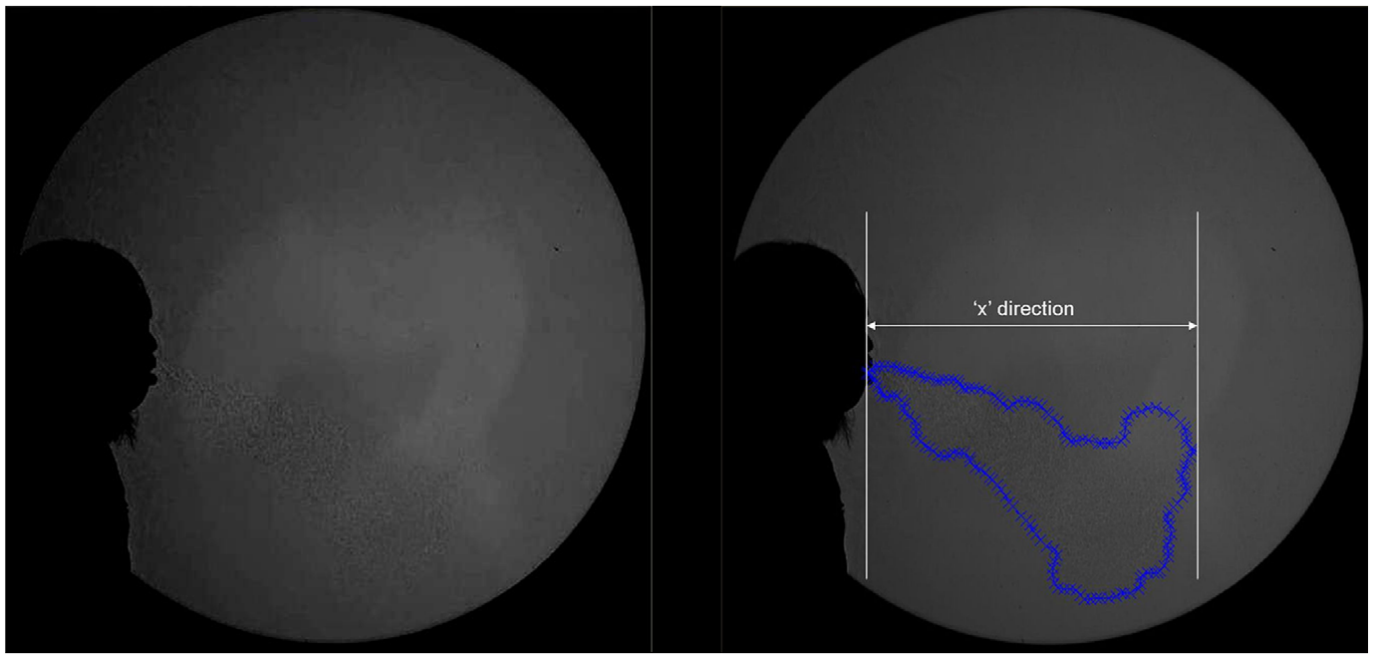
Example of a ‘before’ (A) and ‘after’ (B) digitized shadowgraph image of a human volunteer coughing. These types of images were used to obtain the cough dynamic parameters shown in Figure 3. The blue crosses in image B represent the (x,y) coordinates of the digitized airflow boundary at that point, as seen by one of the independent observers. The software algorithm measured the resolved detectable distance in the ‘x’ direction (B), as this was the clinically important parameter reflecting the horizontal propagation distance of the cough. It also measured the maximum detectable 2-D projected area resolved in the horizontal direction of the cough as seen in the side-on view of the shadowgraph visualization of the cough aerosol.

To digitize the captured images, a software tool, Engauge Digitizer was used (freely available from: http://sourceforge.net/projects/digitizer/). This software allows various points on successive, consecutive image files to be converted to x-y coordinates, when manually selected (e.g. by using a computer mouse) (Figure 2). This cough plume perimeter x-y data was analyzed and plotted using a combination of C++ and Matlab codes (Matlab v.6.5, MathWorks, Natick, MA, USA; http://www.mathworks.com/products/matlab/index.html). The maximum x distance in each frame was calculated by searching the dataset for the point which had the greatest horizontal displacement. The area of the cough plume was calculated by numerically integrating around the cough plume perimeter. The frontal horizontal velocity plot was calculated using the horizontal displacement values vs. time. As velocity is highly sensitive to small changes in displacement, it was decided that the usefulness of plotting velocity based on the raw displacement data was unrepresentative of the flow as this was very much dominated by small, subjective, digitizing-dependent features of the flow field. Instead, by applying a smoothing algorithm based on the weighted moving average of the displacement data, a more representative velocity field can be derived from this raw digitized data.

To analyze these images, the digitizing of each volunteer's cough was performed by two independent observers, using full-screen, 17–19 inch flat LCD monitors, at up to 200% magnification, with the observers stepping backwards and forwards between each frame to ensure the continuity of the airflow from the single cough, to digitize its airflow boundaries as accurately as possible.

In addition, video montages were compiled showing each of these volunteers coughing, as well as the free coughs produced by the volunteers, together with the effects of covering the mouth and nose with an arm in order to limit the dissemination of these aerosols.

## Results

Twenty healthy volunteers (10 women, mean age 32.2±12.9 years; 10 men, mean age 25.3±2.5 years) were recruited for the coughing experiments (details shown in Table 1). All the volunteers were ethnically Chinese and were bilingual for both English and Chinese.

**Table 1:** Characteristics of the 20 healthy human volunteers used in the cough imaging.

Case no.	Age	Sex	Height (H)	Weight (W)	Body-mass index (BMI)	Smoker (Y/N)
	(Years)	(M/F)	(m)	(kg)	(BMI = W/H^2^)	(if Y – no. of 20-cigarette packs per week)
1	21	F	1.65	59	21.7	N
2	23	F	1.62	52	19.8	N
3	24	F	1.62	47	17.9	N
4	25	F	1.62	42	16.0	N
5	26	F	1.70	46	15.9	N
6	26	F	1.68	61	21.6	N
7	31	F	1.48	47.4	21.6	N
8	35	F	1.54	59.4	25.0	N
9	55	F	1.5	57.5	25.6	N
10	56	F	1.56	61.5	25.3	N
11	21	M	1.72	79	26.7	Y (6 packs/week)
12	23	M	1.59	57	22.5	N
13	24	M	1.70	63	21.8	N
14	24	M	1.73	65	21.7	N
15	25	M	1.74	63	20.8	N
16	25	M	1.73	63	21.0	N
17	26	M	1.74	70	23.1	N
18	28	M	1.68	58	20.5	Y (14 packs/week)
19	28	M	1.60	60	23.4	N
20	29	M	1.65	55	20.2	N

The individual plots demonstrated a high degree of variability between the volunteers, which was expected given the different ways each of them coughed. The coughs of some these individual volunteers that were used in this analysis can be seen in the accompanying video montage, where a selection of 10 of these volunteers are also shown coughing freely, then into short and long sleeves (Video S1).

**Figure 3:**
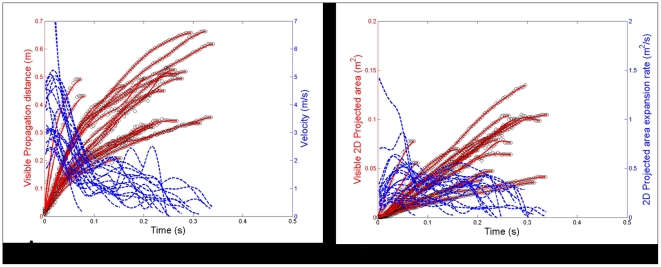
Combined plots of all the coughs produced by the 20 healthy volunteers. These demonstrate the cough airflow dynamic parameters measured in these experiments. **A**: cough ‘propagation distance-velocity-time’; **B:** ‘2-D projected area-expansion rate-time’. The parameters digitized directly from the recorded images (propagation distance and 2-D area) are shown by solid red lines with the actual data points as empty circles, with reference to the left y-axis, labeled with the red font. The derived parameters (velocity and 2-D area expansion rate) are shown by thinner, dotted blue lines, with reference to the right y-axis, labeled with the blue font.

Although multiple ‘coughing bouts’ are shown for each volunteer, only the first ‘cough’ for each of these volunteers was digitized and used in this analysis. When analyzed frame-by-frame, it was relatively easy to distinguish between individual coughs during a ‘bout’ of coughing (i.e. where an individual coughs several times). The results from each of the two observers were compared at various points during the digitizing process and found to be sufficiently similar (within 10% of each other) to allow the two sets of data to be reasonably averaged for the final graphical presentation (Figure 3).

For the 10 females (cases 1–10), their maximum detectable cough propagation distances ranged from 0.16–0.55 m, with maximum derived velocities of 2.2–5.0 m/s, and their maximum detectable 2-D projected areas ranged from 0.010–0.11 m^2^, with maximum derived expansion rates of 0.15–0.55 m^2^/s. For the 10 males (cases 11–20), their maximum detectable cough propagation distances ranged from 0.31–0.64 m, with maximum derived velocities of 3.2–14 m/s, and their maximum detectable 2-D projected areas ranged from 0.04–0.14 m^2^, with maximum derived expansion rates of 0.25–1.4 m^2^/s.

However, despite this intrinsic variability between individual volunteers, for most of these male and female healthy volunteers, the changing values of these cough airflow parameters fell within the limits of the vertical y-axes shown in Figure 3, i.e. for most cases, detectable propagation distances varied between 0–0.6 m, derived velocities vary between 0–6 m/s, detectable 2-D projected areas vary between 0–0.15 m^2^ and derived 2-D projected area expansion rates vary between 0–1.5 m^2^/s. For cases 15 and 20 (both males and neither of whom were smokers), the higher exit velocities of approximately 8.8 m/s and 14 m/s, respectively, may simply be representative of natural variation in this age group as similar values have been previously reported.

For the measured parameters (i.e. propagation distance and 2-D projected area, Figure 3), the general trend is one of increasing over time, which is expected. Some of these values plateau towards the end of their measurable limits, and even appear to decrease slightly, which is probably due to observer variation at the extremes of these detectable limits, when the cough airflow temperature may be equalizing with that of the ambient chamber air.

For the parameters which have been derived from these measured parameters (i.e. cough velocity and 2-D projected area expansion rate, Figure 3) there are more fluctuations as these represent the next time-derivative of the measured parameters, i.e. so any change in the gradient of the propagation distance of 2-D projected area curve will register as a fluctuation in curves of these derived parameters.

It is interesting to note that many cases show a peak cough velocity shortly after the onset of the cough, which is also reflected in a corresponding increase in the 2-D projected area at the same time, which is not unexpected. Finally, as the cough propagation distance and 2-D projected area curves begin to plateau, the curves for the cough velocity and 2-D projected area expansion rate tend towards zero, which is as expected.

Whilst mean and standard deviations of these parameters are easily calculated, we feel that presenting the information summarized in this way would be misleading as each individual cough is quite unique – as can be seen in Video S1.

The cough durations for these 20 cases, as shown in Figure 3, are quite variable. Most lie between 0.20–0.35 s, and none last for more than about 0.35 s, which may represent the maximum duration of visibility for these shadowgraphs, before the exhaled and ambient air temperatures equalized. Some coughs of apparently very short duration can be seen, and re-examination of these video clips revealed that the natural head positions of these volunteers tended to angle their coughs in a downwards direction (and off the bottom edge of the mirror), so limiting the horizontal propagation distance as defined in the digital analysis, but also limiting the number of frames that could be captured before the cough airflow became untraceable as it left the mirror field. Re-recording the coughs with the head position of these volunteers adjusted to allow a more horizontal cough plume to be captured would have extended the distance for which these airflows would have been traceable, but this would not have been the natural posture for these individuals. For some cases, the cough was of very low volume, which may have allowed the temperature of this smaller air mass to decrease more quickly to match that of the ambient air. Hence, these cases exemplify some of the limitations of this naturalistic approach used in this shadowgraph imaging of human coughing.

**Figure 4:**
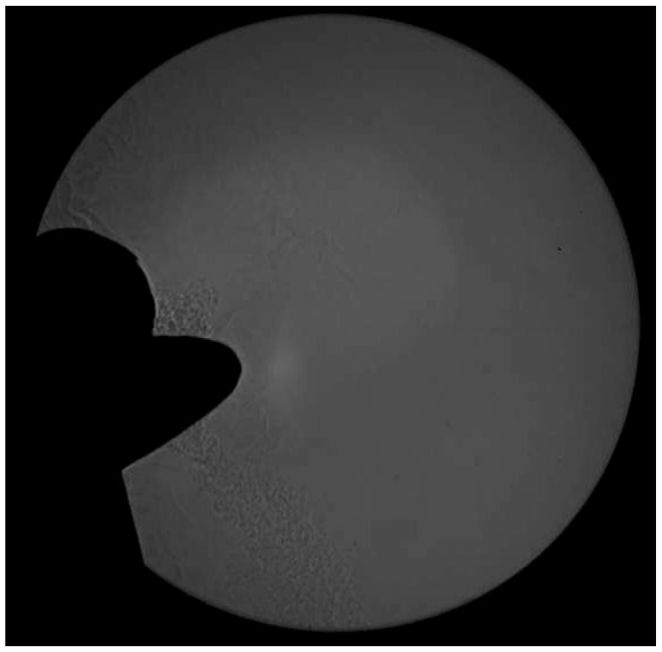
A shadowgraph still image of a cough captured from video. This demonstrates the typical bifurcation of the cough air-stream as a volunteer coughs into his sleeve.

Video S1, demonstrates that the effectiveness of coughing into one's sleeve was also quite variable between individual volunteers, with regard to the degree of blocking of the airflow, depending on how it was performed. Often some form of bifurcation of the cough aerosol was the result (Figure 4). Qualitatively, it appeared to matter less whether short or long sleeves were worn, but more on how the arm was positioned across the mouth and nose.

With only two regular smokers in this cohort of healthy volunteers, it is difficult to draw any definitive conclusions between the effects of smoking and the airflow dynamics of coughing. However, it is perhaps noteworthy that the highest cough propagation distance and 2-D projected area amongst the male volunteers, was produced by a male volunteer who smoked six 20-cigarette packs per week produced may be indicative of the properties of a typically large volume ‘smoker's cough’.

## Discussion

The results shown here for maximum cough velocities agree approximately with previous studies using human volunteers, and estimating their maximum cough velocities using different techniques, though some of these studies use ‘coughed droplets’ rather than purely airflow as their marker of cough velocities.

Particle velocimetry (PIV) has been used by several groups to estimate ranges of cough velocities. Zhu and colleagues used PIV on naturally-produced droplets of saliva to estimate maximum cough velocities of 6–22 m/s and propagation distances of at least 2 m in a calm indoor environment [15]. Using a similar method, Chao and colleagues estimated maximum coughing velocities of 13.2 m/s in a male and 10.2 m/s in a female volunteer [16]. Most recently, using PIV, VanSciver and colleagues reported a range of maximum cough velocities of 1.5–28.8 m/s [17]. A combination of real-time schlieren imaging and PIV gave an estimate of 8/ms for the maximum velocity of a male volunteer's cough [10].

The ranges of these values for maximum cough velocities compare favorably with those obtained using this shadowgraph imaging method, where the human volunteers were able to perform naturally with no movement of postural constraints. Since these maximum cough velocities occurred soon after the cough began, unlike with maximum propagation distances, they are much less affected by the loss of visibility due to rapidly equalizing air temperatures between the exhaled air and the ambient air in the laboratory. Given that the movements and postures of the human volunteers coughing in the PIV experiments were somewhat constrained and unnatural (presumably for experimental design and safety reasons), it might be argued that the results for maximum cough velocities obtained more naturally in this shadowgraph study are a useful validation of those obtained in these more artificial PIV settings.

An interesting study by Gupta et al. [18] examined cough airflow rates (as opposed to velocities per se) using technique of spirometry (a standard clinical investigative tool for patients with chronic respiratory disease) in a cohort of human volunteers who were smokers. However, it is difficult to convert such airflow rates to velocities, without accurate measurements of the changes in shape and size of the mouth opening during coughing. Although Gupta et al. [18] do provide mouth opening measurements, for some reason, they do not use these to provide explicit cough velocity values, making it difficult to compare the outcomes of their studies with these other studies. The physiology of coughing is significantly altered in smokers so these findings may not be applicable to non-smokers [19].

Whilst the overall range of maximum velocities agree relatively well with these previous studies using other methods, a further examination of the graphical plots in Figure 3 reveal some interesting features that may be unexpected not intuitive at first inspection. In many cases there is a peak in velocity soon after the cough leaves the mouth. This may be due to the slight intake of breath before the actual cough airflow acceleration occurs, before the maximum velocity is reached. This has been described elsewhere and appears to be physiological as it follows the changes in subglottic pressure before and during the coughing process [18], [19]. However, in other cases, a further increase in velocity appears to occur even as the overall picture is one of a gradual decrease in airflow velocity. Exactly how and why a high velocity wave-front appears later on in the cough is not clear, but as this phenomenon is relatively common, we believe that purely observer variation cannot be the entire explanation. It could be that, even within one cough action, the airflow moves more quickly in the later half of the cough than in the earlier half of the cough. This could perhaps be due to a change of mouth shape into a more narrow, ‘pursed’ lip shape in the later half of the cough that would tend to accelerate the airflow of the cough past and beyond the slowing moving mass of air generated by the early half of the cough produced using a more open mouth shape. In fact, this dynamic mouth opening behavior during a cough has been documented by Gupta et al [18], and can be seen to some extent in the individual volunteers when coughing in Video S1. Another important physical modulator of the airflow during coughing is the position of the tongue, which may, again, alter the geometry of the mouth through which the air passes during coughing, though this may be more difficult to demonstrate, and no published studies seem to have addressed this aspect, as yet.

These studies raise the interesting question of exactly what should be measured to estimate such velocities, and which measurement would be most relevant for aerosol infection control, e.g. immediate exit velocities (i.e. the speed of the air expelled by the cough at the mouth) or an average velocity measured over a defined time period (and how would such a period be defined)? For example, in a previous study using the shadowgraph technique in one video clip of a volunteer sneezing, a large amount of mucus and saliva was expelled with the sneeze, as shown in that paper's online Supporting Information Video S2 [8], but would measuring the velocity of these droplets really represent the velocity of the sneeze itself? Such droplets move in more ballistic manner [9], and therefore may not necessarily reflect the characteristics of the sneeze itself – if the definition of the sneeze is purely based on the airflow behavior produced. A similar situation may apply to describing cough airflow characteristics. With some of the techniques such as PIV which require a reflective target for obtaining the flow-field measurements, only droplet-related measurements are possible so the measurement method itself may constrain the types of measurement that can be made. Thus, there may be no definitive answer to this question, but as long as researchers define how their cough will be assessed, for the intents and purposes of that particular study, that will be how a cough will be defined.

In this study, using shadowgraph imaging, the cough was treated as purely an airflow phenomenon, with the moving, visible airflow boundaries being used to estimate the distance and area covered by the cough, and frame-by-frame images at high frame-rates allowing instantaneous velocities to be calculated. It is acknowledged that for infections predominantly transmitted by large droplets over shorter distances, the shadowgraph method, as used in this study, may not be optimum as it was designed specifically to examine airflows and therefore, more specifically, the behavior of smaller droplet nuclei that would move with these airstreams more closely. This is simply due to the limitations of this technique, as it was used in these experiments. Visualization of larger droplets is possible with this shadowgraph system – as can be seen in some of the online videos accompanying shown in Tang et al. [8] - but this was not the intent with this study, which was to examine the airflow behavior produced during human coughing.

Given the above, it is important to note that this shadowgraph visualization technique does have significant limitations due to its reliance upon relative differences in temperature (and therefore density) between the exhaled ‘coughed’ air and the surrounding ambient air to visualize the exhaled airflows. As the air leaves the mouth, it rapidly cools as it encounters the much larger volume of colder ambient air, and this cooling effect eventually limits the visibility of this moving ‘cough’ wave-front. Hence, for the cough propagation distances and the 2-D projected area, the plots shown in Figure 3 only cover the airflow dynamic behavior until the airflow boundaries are no longer visible – or the cough goes off the edge of the mirror surface depending on the angle of the cough direction produced by individual volunteers. Whilst the progressive increase in cough propagation distance and 2-D projected area over time might be as expected, the more erratic variation in the cough velocities and 2-D projected area expansion rates may be a result of multiple, overlapping wave-fronts within the ‘coughed’ air mass, pushing the visible edge of the expanding airflow boundaries at different rates over the same period. As only one large mirror and high-speed camera was available for this experimental set-up, a 3-D view was not possible, though this would have been helpful in resolving further details of these multiple, overlapping wave-fronts.

Estimates of the maximum cough velocities and 2-D projected area expansion rate are far less affected by this limitation as the maximum values of these derived parameters occur soon after the cough airflow leaves the mouth when it is still considerably warmer than the ambient air so their airflow boundaries are still very visible. These values compare favorably with previous estimates obtained using other methods, as described earlier [15]–[17].

The CDC recommendation to use the arm or sleeve to block the cough airflow is presumably based on: 1) the impaction and entrapment of larger, more ballistic particles in the substance of the sleeve, so as not to land on any other person or surface (fomite) nearby; 2) the reduction in the velocity of the coughed airflow to limit the distance of dissemination; 3) coughing into their sleeves instead of their hands makes it less likely that any potentially infected mucus will be transferred to other people or surfaces from where it could be picked up others. Considering the first of these concepts in the context of this study, whilst larger droplets moving ballistically are occasionally seen and captured on the shadowgraph images, this technique is mainly aimed at observing and recording the behavior of the smaller (‘droplet nuclei’) that will tend to move more closely with the cough air-stream. Such large droplets will tend to fall out of the air-stream quite rapidly and may not pose a significant risk for longer-distance dissemination, particularly during coughing when the main risk may arise from the larger numbers of smaller droplets that tend to be produced and which may be carried further [20]. In this regard, the observed behavior of the airflow in these shadowgraph images tends to support the second concept described above, i.e. to limit the distance propagated by potentially infectious ‘droplet nuclei’ carried in these coughed aerosols. In addition, some redirection of these aerosols (into the bifurcations – Figure 4) is also seen – which may or may not be advantageous, depending on the position and proximity of people nearby (e.g. when standing on a subway or bus). No attempt was made to digitize the boundaries of the airflows arising from coughing into one's sleeve because it was considered that the resulting airflows were too diverse to make this useful in the context of the other variable that was difficult to quantify, i.e. that positioning of the arm or sleeve of the volunteer. On the third concept above, whilst coughing into one's sleeve certainly avoids the contamination of one's hands, unsightly stains from drying mucus or saliva on one's sleeves may not be desirable, especially when expensive clothing and/or a high-profile occupation is involved. In summary, it is likely that the effectiveness of this CDC-recommended intervention is determined by the careful positioning of the mouth and nose within the material of the sleeve, as is demonstrated by some of the volunteers shown in this video, though this may not be practically possible in many situations in everyday life.

With regard to the effect of smoking, further larger studies containing more healthy volunteers who smoke to varying degrees may reveal a more definitive relationship between non-smokers and smokers with regard to any significant differences in their cough airflow dynamics. Finally, the coughs analyzed here are all purely voluntary coughs, where the volunteers were asked to cough on cue in an experimental environment. The airflow dynamics of coughs which arise naturally (e.g. as a result of a respiratory infection or exposure to an irritant or allergen) or coughing in different situations (e.g. the polite social coughs that may be produced in embarrassing situations) maybe exhibit different airflow dynamics and further studies will be required to characterize these more accurately.

In summary, this study adds to the body of data characterizing the airflow dynamics of voluntary coughs from healthy human volunteers. The main advantage of this shadowgraph approach is that it allows the human volunteers to perform and move naturally during coughing with no restraints. This is in contrast to the PIV studies in which the movement of the human volunteers are usually unnaturally constrained to some extent, for safety reasons. Hence, the results from this shadowgraph imaging method might also be considered as a valuable validation of these PIV studies.

This airflow dynamical data in combination with data from other researchers investigating exhaled or expelled droplet characteristics from human volunteers will be useful to understand the risk that coughing may pose for the transmission of airborne infectious agents, and therefore to improve aerosol infection control interventions in healthcare and community environments.

## Supporting Information

Video S1 **A series of video clips showing healthy volunteers coughing.** Ten healthy (4 females) volunteers (age 21–28 years) coughing freely, then into their sleeves to demonstrate the effectiveness of this intervention (as recommended by the US CDC: http://www.cdc.gov/flu/protect/covercough.htm) in limiting the dissemination of the cough aerosol. The first 5 volunteers cough into short sleeves, and the second 5 volunteers cough into long sleeves. It can be seen that in many cases, the cough plume tends to bifurcate into separate streams passing above and below the intervening arm, in some cases with little noticeable loss of momentum. This effectiveness of this intervention is necessarily subject to the degree with which the individual has time to carefully cover the nose and mouth completely with the sleeve, which may not be always possible in various everyday situations.
(WMV)
